# ICP6 Prevents RIP1 Activation to Hinder Necroptosis Signaling

**DOI:** 10.3389/fcell.2020.595253

**Published:** 2020-10-30

**Authors:** Hong Hu, Guoxiang Wu, Zhaoqian Shu, Dandan Yu, Ning Nan, Feiyang Yuan, Xiaoyan Liu, Huayi Wang

**Affiliations:** ^1^School of Life Science and Technology, ShanghaiTech University, Shanghai, China; ^2^CAS Center for Excellence in Molecular Cell Science, Shanghai Institute of Biochemistry and Cell Biology, Chinese Academy of Sciences, Shanghai, China; ^3^University of Chinese Academy of Sciences, Beijing, China

**Keywords:** necrosome, RIP3, necroptosis, RIP1, ICP6

## Abstract

Necroptosis is a type of programmed necrosis which depends on the activation of receptor-interacting protein kinase 3 (RIP3). Herpes simplex virus type 1 (HSV-1) is known to block necroptosis by the viral protein ICP6 in human cells, but its specific inhibitory mechanism is not fully understood. Here we reported that ICP6 could promote rather than suppress the formation of necrosome, the necroptosis signaling complex containing RIP3 and upstream regulator receptor-interacting protein kinase 1 (RIP1), but blocked RIP3 activation. Moreover, ICP6 could reduce the necroptosis-specific auto-phosphorylation of RIP1 regardless of the presence of RIP3. These results indicate that ICP6 block necroptosis through preventing RIP1 activation dependent signal transduction in necrosome.

## Introduction

Under the selection pressure, the pathogen and the host are engaging in a long-term defense and counter-defense war (Mocarski et al., 2012; Mossman and Weller, 2015). Cell death play an important role in the mammalian host defense system when pathogens invade (Wang X. et al., 2014). There are two classical forms of programmed cell death: apoptosis and programmed necrosis. Apoptosis is characterized by cell shrinkage, chromosomal DNA fragmentation, and the formation of apoptotic bodies (Kerr et al., 1972). But the cell membrane is intact and does not cause inflammation. While necrosis is characterized by swelling of cells and organelles, rupturing cell membranes and releasing of contents and triggering inflammation and immune response (Festjens et al., 2006). Apoptosis can be stimulated by the intrinsic or extrinsic signaling pathways which depend on caspase-9 and caspase-8, respectively (Li et al., 1997; Du et al., 2000; Verhagen et al., 2000; Peter and Krammer, 2003). The extrinsic apoptosis pathway is initiated by various cytokines including tumor necrosis factor (TNF). Interestingly, TNF can also induce cell necroptosis, a type of programmed necrosis when caspase-8 is inhibited (Vercammen et al., 1998). The signaling of necroptosis is dependent of a protein complex called necrosome, which contains receptor-interacting protein kinase 1 (RIP1), receptor-interacting protein kinase 3 (RIP3), and mixed lineage kinase domain-like protein (MLKL) (Holler et al., 2000; Degterev et al., 2005; Cho et al., 2009; He et al., 2009; Zhang et al., 2009; Sun et al., 2012; Zhao et al., 2012). The necrosome is initiated with the binding of RIP1-RIP3 through their RIP homotypic interaction motif (RHIM) (Sun et al., 2002). In necrosome, the RIP1 and RIP3 is sequentially activated (He et al., 2009). Then MLKL is recruited and phosphorylated by RIP3, which induce MLKL to form oligomers and migrate to the membrane (Wang H. et al., 2014). Finally, MLKL destroys the integrity of the cell membrane and causes cell rupture (Su et al., 2014; Wang H. et al., 2014).

Pathogens and hosts are constantly evolving for survival. Upon virus infection, the conventional strategy of host is to initiate apoptosis of infected cells, thus limiting the amplification and spread of the virus (Chan et al., 2015; Mossman and Weller, 2015). However, many viruses have evolved counteractive effectors such as the inhibitors of caspase (IAPs) to inhibit apoptosis (Kaiser et al., 2013). Usually, necroptosis signaling is supervised by caspase-8 which can cleavage RIP1 and RIP3 to prevent necrosome formation (Lin et al., 1999; Feng et al., 2007). Therefore, the expression of viral IAPs will sensitize infected cells to necroptosis. Consisted with that, RIP3-deficent mice are developmental normal but exhibited severely impaired virus-induced necrosis and restriction of viral amplification (Cho et al., 2009). Necroptosis is regarded as an alternative defense pathway in the infected cells, when apoptosis is inhibited (Chan et al., 2015; Mossman and Weller, 2015). Although necroptosis is effective to inhibit many virus amplifications, some viruses such as herpes simplex virus type 1 (HSV-1) and murine cytomegalovirus (MCMV) can block necroptosis signaling by special viral effectors, ICP6 and M45 (Upton et al., 2010; Guo et al., 2015; Huang et al., 2015; Yu et al., 2016). The ICP6 (from HSV-1) and M45 (from MCMV) are homologous, with similar domain structures, which contain an N-terminal RHIM domain and a C-terminal ribonucleotide reductase (RNR) large subunit (R1)–homology domain (Lembo and Brune, 2009). The inhibitory function of M45 in necroptosis pathway is solely dependent on its RHIM, which binds with RIP3-RHIM and form hetero-amyloids to prevent RIP3 activation by disturbing inter-filament assembly of RIP3-RHIM (Hu et al., 2020). But the ICP6-RHIM alone do not block RIP3 activation, suggesting a different mechanism to disrupt necroptosis signaling (Hu et al., 2020).

In this study, we confirmed the previous finding that both the N-terminal RHIM and C-terminal R1 domains of ICP6 are required for its inhibitory function. And ICP6 block necroptosis is not due to prevent RIP1-RIP3 binding and necrosome initiation. ICP6 could participate in necrosome formation with interaction with both RIP1 and RIP3 upon necroptosis induction. Interestingly, ICP6 can attenuate necroptosis signal induced auto-phosphorylation of RIP1 regardless of the presence of RIP3, suggesting ICP6 hinder necroptosis signaling through preventing RIP1 activation.

## Materials and Methods

### Reagents and Antibodies

The Z-VAD and Smac mimetic were used as described previously (Wang H. et al., 2014). Recombinant TNF was purified in our laboratory, AP20187 (HY-13992) was purchased from MCE. ICP6 polyclonal antibodies can be obtained by injecting His-ICP6 (1-177 amino acids) expressed in *E. coli* into rabbits. The following antibodies were used in this study: anti-Flag M2 (F3165), anti-Myc (SAB4700448) (Sigma); anti-RIP1 (610459) (BD Biosciences); anti-RIP1 (D94C12), anti-phospho-RIP1 (65746S) and anti-hRIP3 (13526S) (Cell Signaling Technology); anti-FKBP (ab108420) (Abcam); and anti-actin (PM053-7) (MBL).

### Cell Survival Assay

Drugs were diluted and added into cell culture plate. Necroptosis was induced by adding the final concentrations of 10 ng/ml TNF-α (T), 100 nM Smac mimetics (S), and 20 μM Z-VAD (Z). Cell survival assay was performed by using the CellTiter-Glo Luminescent Cell viability Assay Kit and performed according to the manufacturer’s instructions. Luminescence was recorded with an EnSpire Multimode Plate Reader from PerkinElmer.

### Cell Culture and Stable Cell Lines

HEK293T cells, HeLa cells, and HT-29 human colon cancer cells were obtained from cell bank of CAS (Shanghai). HeLa-RIP3 (HeLa with exogenous Flag-tagged RIP3 expression) cells were established as described previously (He et al., 2009; Sun et al., 2012; Wang H. et al., 2014). HT-29 (*RIP3*-KO) cells were generated by using the CRISPR/Cas9 editing technique. KO cells were identified by target site sequencing. Cells would stably express related proteins after lentivirus infection. All cells were cultured in DMEM/L-glutamine without sodium pyruvate (HyClone). All media were supplemented with 10% Foundation^TM^ FBS (Gemini) and 100 units/ml penicillin/streptomycin (HyClone). All cells were cultured at 37°C, 5% CO_2_, and the standard PCR method was negative for mycoplasma. EZ transfection (Shanghai Life iLab Biotech Co., Ltd.) was used for cell transfection according to the instructions.

### Plasmids and Molecular Cloning

ICP6 cDNA was amplified by PCR from a reverse transcription cDNA library. Standard PCR and cloning methods were used to clone the full-length or mutated cDNAs of ICP6 into the lentiviral vector pCDH-CMV-MCS-EF1-copGFP (Addgene). All plasmids have been verified by DNA sequencing. The detailed information of primer sequence can be provided upon request.

### Lentivirus Preparation and Infection

For lentivirus production, HEK293T cells were transfected with lentiviral vectors (pCDH-CMV-MCS-EF1-copGFP/copRFP) and virus packing plasmids (psPAX2 and pMD2.g, Addgene) by using EZ transfection reagents (Shanghai Life-iLab Biotech Co., Ltd.). After 48 h, the virus-containing medium was collected and added to the cells according to the instructions and the final concentration of polybrene was 10 μg/ml. After 24 h, the infection medium was replaced with fresh medium.

### CRISPR/Cas9

The Cas9-target sites are as follows: human RIP3: 5′-GACAGGGTCCGGGGAGCCAG-3′ and 5′-GCAAGCCGGGCCTGAGACTCC-3′. The Cas9-target sites were cloned into the PX330 (Addgene) vector. All plasmids were verified by DNA sequencing. The details of the primer sequences are available upon request.

### Immunoprecipitation and Immunoblotting

Cells were cultured on 10 cm dishes and grown to confluence. Cells at 90% confluence were washed once with DPBS and harvested and centrifuged at 900 rpm for 3 min. The harvested cells were lysed by lysis buffer [25 mM HEPES-NaOH (pH 7.5), 150 mM NaCl, 1% Triton, 10% glycerol, and complete protease inhibitor (Roche) and phosphatase inhibitor (Roche) cocktails] on ice for 30 min. Cell lysates were centrifuged at 15000 rpm at 4°C for 10 min. The soluble fraction was collected. The protein solution were incubated overnight with anti-Flag or anti-Myc magnetic beads at 4°C. The beads were washed three times with lysis buffer. Finally, the samples were subjected to western blotting analysis.

### Immunofluorescence Staining and Confocal Microscopy

HeLa-RIP3 and HT29 cells infected with lentivirus were plated on the coverslip overnight and then treated as shown. The cells were washed once with PBS, and then fixed with 4% paraformaldehyde (PFA). The primary antibody (Flag, RIP1 or ICP6) was incubated. Then, the cells were incubated with goat anti-mouse IgG labeled with Alexa Fluor 555 (Invitrogen) for RIP1 or 633 (Invitrogen) for Flag and goat anti-rabbit IgG labeled with Alexa Fluor 647 (Invitrogen) for ICP6. Finally, the nuclei were stained with DAPI (Southern Biotech) and using the same settings for image capture and processing on the Zeiss LSM 710 laser scanning confocal microscope (63×).

### Quantification and Statistical Analysis

All cell survival data are shown as the mean ± standard deviation (SD) of duplicate wells, and similar results were obtained from at least three independent experiments.

## Results

### The R1 Domain of ICP6 Is Unique and Essential in Inhibiting Necroptosis

**FIGURE 1:**
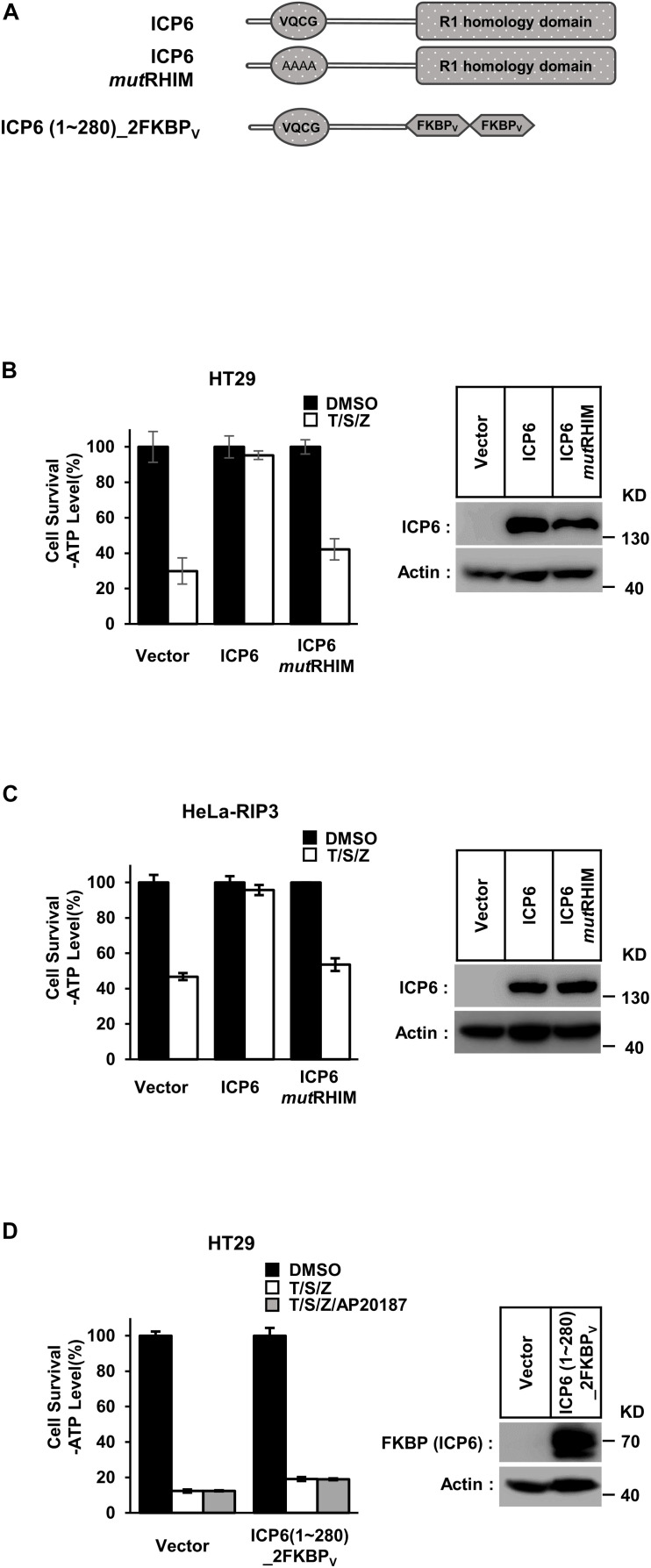
Either RHIM or R1 domain of ICP6 is indispensable for necroptosis blockage. **(A)** Schematic representation of full-length ICP6 with or without RHIM mutations (upper). ICP6 N-terminal portion (1-280) was fused with tandem FKBPv domain at its C-terminus (lower). **(B,C)** The RHIM of ICP6 is essential for inhibition of necroptosis. The HT29 and HeLa-RIP3 (HeLa with exogenous Flag-tagged RIP3 expression) cells stably expressing ICP6 protein by lentivirus infection were stimulated with T/S/Z for 10 h. The number of surviving cells were analyzed by measuring ATP levels using CellTiter-Glo kit **(left)**. The data are represented as the mean ± standard deviation (SD) from at least three independent experiments. Abbreviations are as follows: T, TNF-α; S, Smac mimetic; Z, Z-VAD. The final concentrations of 10 ng/ml TNF-α, 100 nM Smac mimetic, and 20 μM Z-VAD were used. Identical concentrations of these necroptosis-inducing agents were used in subsequent experiments unless otherwise stated. The untreated cells were harvested and whole cell extracts were prepared and normalized to the same concentration. Aliquots of 20 μg whole-cell lysates were subjected to SDS-PAGE followed by western blot analysis of ICP6 and β-Actin which is shown as a loading control **(right)**. **(D)** The R1-homology domain of ICP6 has a unique function in inhibiting necroptosis. HT29 cells were infected with lentiviruses encoding ICP6 (1-280)_2FKBPv and treated with indicated stimuli. The number of surviving cells was determined by measuring ATP levels **(left)**. The ICP6 (1-280)_2FKBPv expression level was measured by western blot analysis **(right)**.

According to previous reports, full-length ICP6 can effectively inhibit necroptosis in human cells, but induce mouse cell necroptosis (Huang et al., 2015). It requires the structural integrity of both RHIM and R1 domain. First, we constructed wild-type and RHIM-mutated ICP6 to confirm the requirement of intact ICP6-RHIM (Figure 1A, upper). In either human colon cancer HT29 cells with endogenously RIP3 expression or HeLa-RIP3 cells with exogenously expressed Flag-tagged RIP3, necroptosis could be induced by TNF plus a pan-caspase inhibitor Z-VAD-FMK (Z-VAD) and Smac-mimic small molecule compound (Smac-mimic). We confirmed that the expression of wild-type rather than RHIM-mutant of ICP6 could inhibit necroptosis in HT-29 and HeLa-RIP3 cells (Figures 1B,C). The importance of R1 domain of ICP6 has also been suggested by previous studies (Guo et al., 2015). And the function of R1 domain in ICP6-induced mouse cell necroptosis depends on the oligomeric state of R1 domain (Huang et al., 2015). So that the function of ICP6-R1 in mouse cells could be mimic by chemical (AP20187)-induced oligomerization of the tandem FKBPv domain. To investigate if oligomeric state of R1 domain could contribute its necroptosis inhibitory function in human cells. We constructed ICP6 with the R1 domain replaced by tandem FKBPv domain [ICP6 (1∼280)_2FKBP_V_] (Figure 1A, lower). In HT-29 cells even if the oligomerization is induced by AP20187 (Figure 1D) and it also doesn’t inhibition necroptosis. These results indicate that the R1 domain is essential for the inhibitory function of ICP6.

**FIGURE 2:**
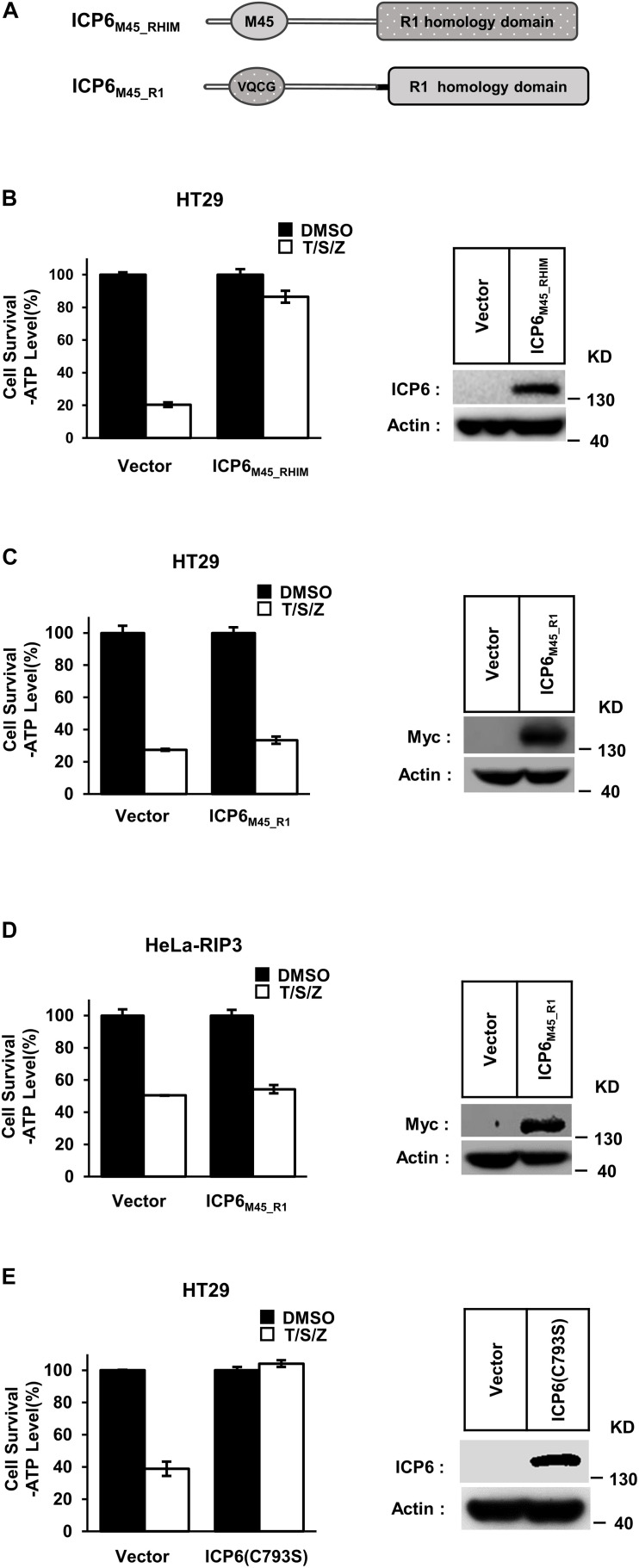
The R1 domain of ICP6 is unique in inhibiting necroptosis. **(A)** Schematic representation of chimaeric ICP6 containing M45 RHIM and R1-homology domain. **(B)** The chimaeric ICP6 containing M45 RHIM has no effect on the inhibition of necroptosis. Necroptosis was induced by T/S/Z for 10 h. Then the cells were lysed and subjected to western blotting analysis using the indicated antibodies. **(C,D)** The R1 homology domain is important to ICP6 function. The HT29 **(C)** and Hela-RIP3 **(D)** cells with indicated lentivirus infection were treated with T/S/Z for 10 h. Cell viability was determined by measuring ATP levels **(left)**. The data are represented as the mean ± SD of duplicate wells. The expression level of ICP6_M45_____R1_ was measured by western blot analysis **(right)**. **(E)** The ICP6 (C793S) has no effect on the inhibition of necroptosis. Necroptosis was induced by T/S/Z for 10 h. Then the cells were lysed and subjected to western blotting analysis using the indicated antibodies.

Different from ICP6, the inhibitory role of M45 in necroptosis signaling does not require R1 domain of M45. But it still needs to reveal that if the function of R1 in necroptosis repression is universal in different R1 domains. We then constructed the chimaeric ICP6 containing M45 RHIM or R1 domain (ICP6_M45_RHIM_ and ICP6_M45_____R1_) (Figure 2A). We found that ICP6_M45_RHIM_ could effectively inhibit necroptosis in HT29 cells (Figure 2B). This suggests that the RHIM domain of ICP6 can be replaced by the RHIM domain of M45 in inhibiting necroptosis. In ICP6_M45_____R1_ expression HT29 or HeLa-RIP3 cells, TNF-induced necroptosis was not inhibited (Figures 2C,D). It suggested again that the oligomeric state of R1 domain did not contribute to necroptosis inhibition since both M45-R1 and ICP6-R1 could form oligomers. Besides that, it also indicated that the R1 domain of ICP6 is special in necroptosis inhibition. The difference of R1 domain between ICP6 and M45 is that the ICP6-R1 has but M45-R1 loses the ribonucleotide reductase activity. To investigate how the enzyme activity of ICP6-R1 is necessary for necroptosis inhibition. The enzyme-dead form of ICP6 mutant (C793S) was introduced to HT-29 cells. But it showed that the mutant form of ICP6 still block necroptosis efficiently (Figure 2E). It suggested the function of ICP6-R1 do not require its enzyme activity.

### ICP6 Promotes Necrosome Initiation but Block Necrosome Maturation

**FIGURE 3:**
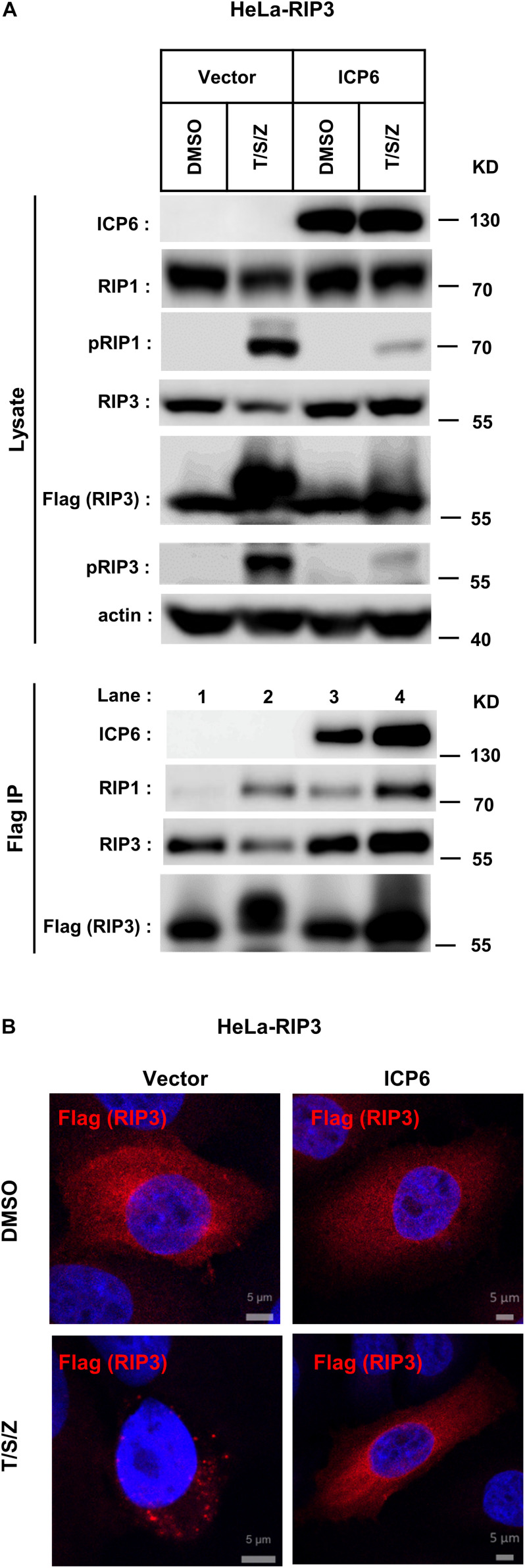
ICP6 promotes necrosome initiation but block necrosome maturation. **(A)** HeLa-RIP3 cells (HeLa with exogenous Flag-tagged RIP3 expression) infected with empty virus (Vector) or lentiviral virus encoding ICP6 were cultured in the presence of T/S/Z for 6 h. Whole-cell lysates were subjected to immunoprecipitation with anti-Flag M2 beads. The total cell lysates and immunoprecipitates were immunoblotted with the indicated antibodies. **(B)** ICP6 inhibits the formation of RIP3 puncta. HeLa-RIP3 cells infected with empty virus (Vector) or lentiviral virus encoding ICP6 were stimulated as indicated for 6 h, and then immunostained for Flag and counterstained with DAPI. The distribution of Flag-RIP3 (Alexa Fluor 633 dye, red) was detected by immunofluorescence as described in “Materials and Methods.” The scale bar represents 5 μm.

**FIGURE 4:**
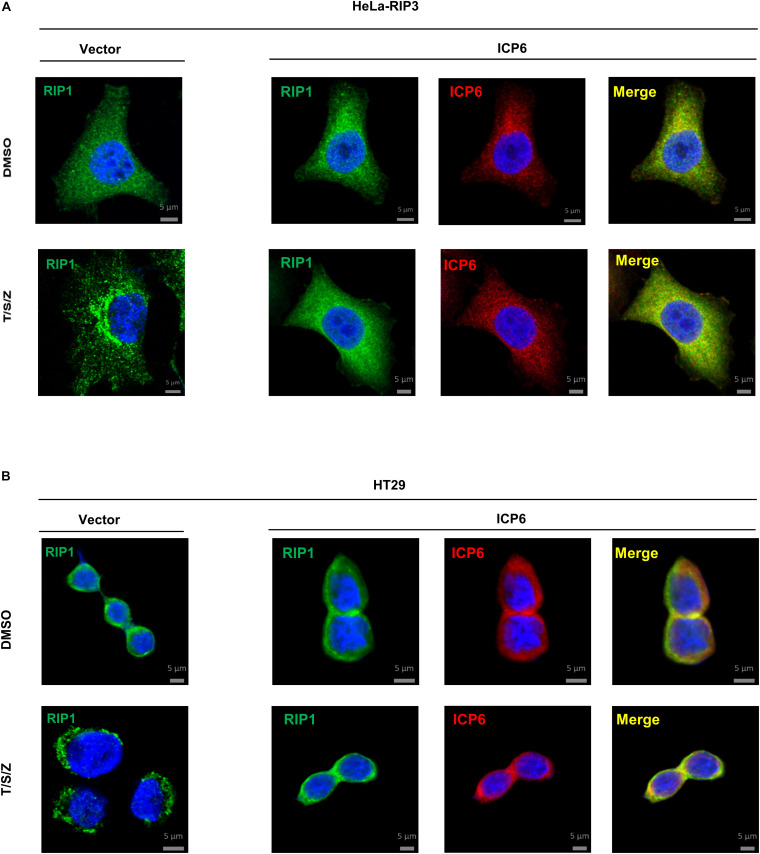
ICP6 inhibits the formation of RIP1 puncta. **(A,B)** HeLa-RIP3 and HT29 cells infected with empty virus (Vector) or lentiviral virus encoding ICP6 were stimulated as indicated for 6 h, and then immunostained for RIP1, ICP6, and counterstained with DAPI. The distribution of RIP1 (Alexa Fluor 555 dye, green) and ICP6 (Alexa Fluor 647 dye, red) were detected by immunofluorescence as described in “Materials and Methods”. The scale bar represents 5 μm.

It is well known that the necrosome formed by RIP1 and RIP3 is essential for TNF-induced necroptosis. And this necroptosis signal complex is initiated by RHIM-RHIM interactions between RIP1 and RIP3. Since ICP6-RHIM is required for its function, confirmed by our data shown that RHIM-mutated ICP6 lost the ability to inhibit TSZ-induced necroptosis (Figures 1B,C), We proposed that RIP1-RIP3 interactions maybe disturbed by ICP6-RHIM, so that it inhibited the necrosome formation and necroptosis signal transduction. To verify that, we performed co-immunoprecipitation by Flag-tagged RIP3 in HeLa-RIP3 cells. It showed that the protein level of co-immunoprecipitated RIP1 is not reduced but increased in TSZ treated cells with ICP6 expression (Figure 3A, lane 3 vs. lane 4). Moreover, compared with empty vector, the presence of ICP6 enhanced the interaction between RIP1 and RIP3 in the DMSO-treated cells (Figure 3A, lane 1 vs. lane 3). Different from the binding of RIP1-RIP3 which depended on necroptosis stimuli (lane 2), ICP6 could bind with RIP3 in normal conditions (lane 3), but was also enhanced upon necroptosis induction (lane 4). Altogether, these data suggested that ICP6 did not disturb but promoted RIP1-RIP3 binding and necrosome initiation. Then, the effect of ICP6 at step of necrosome maturation was investigated. The necrosome maturation is featured by RIP3 self-assembly as puncta in necrotic cells (He et al., 2009). As shown that TSZ-induced RIP3 puncta were observed in control cells transfected with empty vector. However, in ICP6-expressing cells, RIP3 remained uniformly diffuse in the cytosol, no matter whether necroptosis was induced or not (Figure 3B). The co-immunoprecipitation results showed that necrosomes are made of RIP1-RIP3 or RIP1-RIP3-ICP6 (when ICP6 was expressed) complexes (Figure 3A). We then using RIP1 antibody to detect the necrosome puncta in both Hela-RIP3 and HT-29 cells, the RIP1 puncta appeared after necroptosis induction, and ICP6 expression blocked the T/S/Z-induced RIP1 puncta formation (Figures 4A,B). Besides that, the RIP1 and ICP6 signals partially overlapped and were diffused in cytosol (Figures 4A,B). It confirmed the necrosome puncta only reflect the matured necrosome. So that, ICP6 promotes necrosome initiation but block necrosome maturation. The premature state of necrosome was featured as decreased auto-phosphorylation of RIP1 and RIP3 (Figure 3A, lane 2 vs. lane 4). Since the RIP1 activation (indicted by auto-phosphorylation) is upstream of RIP3 activation, it suggested ICP6 blocks necroptosis signaling by targeting on RIP1.

### The Inhibition of RIP1 Auto-Phosphorylation by ICP6 Is RIP3-Independent

**FIGURE 5:**
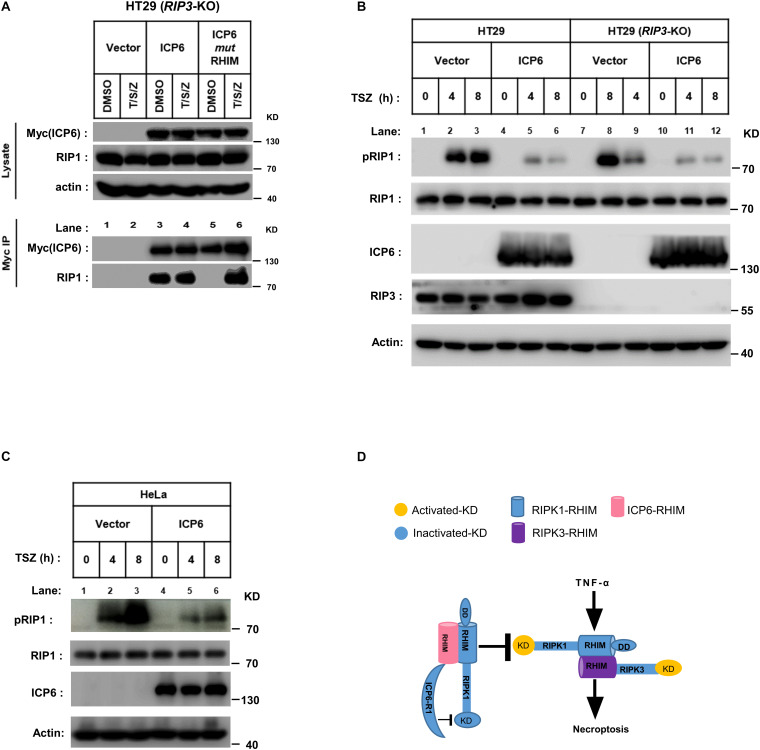
The inhibition of RIP1 auto-phosphorylation by ICP6 is *RIP3*-independent. **(A)** HT29 (*RIP3*-KO) cells were infected with empty virus (Vector) or lentiviral virus encoding Myc-tagged ICP6. Then these cells were treated with T/S/Z for 6 h. Whole-cell lysates were subjected to immunoprecipitation with anti-Myc. The total cell lysates and immunoprecipitates were analyzed by western blot with the indicated antibodies. **(B,C)** ICP6 reduces the phosphorylation of RIP1. HT29, HT29 (*RIP3*-KO) **(B)** and HeLa **(C)** cells with indicated lentivirus infection were treated with T/S/Z for indicated time. The cells were then harvested, and the whole-cell extracts were analyzed by western blotting to check the RIP1 activation with anti-phospho-hRIP1 (S166). β-Actin and total RIP1 were shown as loading control. **(D)** The proposed mechanism of ICP6 inhibited necroptosis. KD, kinase domain; RHIM, RIP homotypic interaction motifs; DD, death domain; R1, C-terminal R1 homology domain of ICP6. Cells with necroptosis induction (TNF) undergo RIP1-*RIP3* interaction and kinase activation which are indispensable for necrosome maturation and cell necroptosis. ICP6 block necroptosis through ICP6-RIP1/3 RHIM domain interaction, then the ICP6-R1 could block RIP1 kinase activation to necrosome maturation.

ICP6 does not inhibit the formation of RIP1-RIP3 necrosome but invade into this necroptosis signal complex. Then we want to know if the blockage of RIP1 activation by ICP6 needs RIP3. We first examined the binding between ICP6 and RIP1 in HT29 cells with *RIP3* knockout. The co-immunoprecipitation results showed that wild-type rather than RHIM mutant of ICP6 bind with RIP1 in DMSO treated control cells (Figure 5A, lane 3 vs. lane 5). Interestingly, both wild-type and RHIM mutant form of ICP6 interact with RIP1 upon necroptosis induction (Figure 5A, lane 4 vs. lane 6). Thus, these results demonstrated that ICP6 could directly interact with RIP1 in absence of RIP3. Then, the inhibition of RIP1 auto-phosphorylation by ICP6 was checked in *Rip3* knockout HT29 cells. The results showed that whether RIP3 is present or not, the increment of RIP1 auto-phosphorylation level was attenuated when ICP6 was expressed (Figure 5B). Similar results were obtained in HeLa cells which are absent of endogenous RIP3-expression (Figure 5C). These results suggested the necroptosis signal induced RIP1 activation was attenuated by ICP6. So, we proposed the model of ICP6 blocking necroptosis. ICP6 binds with both RIP1 and RIP3 through RHIM-RHIM interaction to promote necrosome initiation. But in necrosome, ICP6 (probably using its R1 domain) block RIP1 activation, so that the ICP6 containing necrosome could not maturate and process cell necroptosis (Figure 5D).

## Discussion

Receptor-interacting protein kinase 1 (RIP1) is composed of kinase domain, RHIM domain and death domain. In TNF-induced cell death pathways, the kinase activity is required for both RIP1 dependent apoptosis (RDA) and necroptosis (Yuan et al., 2019). Our present work reveals that ICP6 could attenuate necroptosis signal induced RIP1 activation (Figures 5B,C), so that the necroptosis signaling is blocked by ICP6 on RIP1. Interestingly, the inhibitory function of ICP6 needs both of RHIM domain and R1 domain. The RHIM domain of ICP6 is responsible for efficient binding to RIP1 (Figure 5A). For R1 domain of ICP6, our data ruled out the possibilities that the enzymatic activity or oligomeric state of R1 domain contribute necroptosis inhibition (Figures 1D, 2E). Therefore, how R1 domain of ICP6 functions in necroptosis blockage still needed to be studied.

ICP6 is encoded by a viral gene HSV-1. It is known that HSV-1 is a human-hosted virus belonging to the alpha herpesvirus subfamily. Infection with HSV-1 mainly causes herpes, and sporadic encephalitis for severe cases (Steiner and Benninger, 2013). Other symptoms by HSV-1 infection were reported, such as a latent infection in the peripheral nervous system of the host. It is reported that after infection with HSV, the immune system is destroyed and ulcers in the affected area increase the probability and risk of HIV infection (van Velzen et al., 2013). In epithelial cells, HSV-1 can induce the secretion of TNFα, IL-1β and other inflammatory factors, that is, the inflammatory response participates in the host’s resistance to pathogen infection (Muscolino et al., 2020). RIP1 plays a very important role in inflammation through NF-κB or p38 MAPK pathways which is independent of its kinase activity. Our data showed that ICP6 attenuate RIP1 activation, and it is interesting to study if other functions of RIP1 are affected by HSV-1 infection or ICP6 expression.

## Data Availability Statement

The raw data supporting the conclusions of this article will be made available by the authors, without undue reservation.

## Author Contributions

HH and HW conceived and designed the experiments, drafted the manuscript, discussed the results, and edited the manuscript. HH performed and analyzed most of the experiments. GW contributed to the performance of the experiments. ZS, DY, NN, FY, and XL generated essential tools and reagents for the study. All authors contributed to the article and approved the submitted version.

## Conflict of Interest

The authors declare that the research was conducted in the absence of any commercial or financial relationships that could be construed as a potential conflict of interest.
